# RuvB-Like Protein 2 Interacts with the NS1 Protein of Influenza A Virus and Affects Apoptosis That Is Counterbalanced by Type I Interferons

**DOI:** 10.3390/v13061038

**Published:** 2021-05-31

**Authors:** Yimeng Wang, Jianhong Zhou, Samuel G. Mackintosh, Yuchun Du

**Affiliations:** 1 Department of Biological Sciences, University of Arkansas, Fayetteville, AR 72701, USA; yimengwang@revaccbio.com (Y.W.); jxz011@uark.edu (J.Z.); 2 Department of Biochemistry and Molecular Biology, University of Arkansas for Medical Sciences, Little Rock, AR 72205, USA; mackintoshsamuelg@uams.edu

**Keywords:** influenza A virus, NS1, RuvBL1, RuvBL2, apoptosis, interferon

## Abstract

The NS1 protein of influenza A virus (IAV) plays important roles in viral pathogenesis and host immune response. Through a proteomic approach, we have identified RuvB-like proteins 1 and 2 (RuvBL1 and RuvBL2) as interacting partners of the NS1 protein of IAVs. Infection of human lung A549 cells with A/PR/8/34 (PR8) virus resulted in reductions in the protein levels of RuvBL2 but not RuvBL1. Further studies with RuvBL2 demonstrated that the NS1-RuvBL2 interaction is RNA-independent, and RuvBL2 binds the RNA-binding domain of the NS1. Infection of interferon (IFN)-deficient Vero cells with wild-type or delNS1 PR8 virus reduced RuvBL2 protein levels and induced apoptosis; delNS1 virus caused more reductions in RuvBL2 protein levels and induced more apoptosis than did wild-type virus. Knockdown of RuvBL2 by siRNAs induced apoptosis and overexpression of RuvBL2 resulted in increased resistance to infection-induced apoptosis in Vero cells. These results suggest that a non-NS1 viral element or elements induce apoptosis by suppressing RuvBL2 protein levels, and the NS1 inhibits the non-NS1 viral element-induced apoptosis by maintaining RuvBL2 abundance in infected cells in the absence of IFN influence. In contrast to Vero cells, infection of IFN-competent A549 cells with PR8 virus caused reductions in RuvBL2 protein levels but did not induce apoptosis. Concomitantly, pretreatment of Vero cells with a recombinant IFN resulted in resistance to infection-induced apoptosis. These results demonstrate that the infection-induced, RuvBL2-regulated apoptosis in infected cells is counterbalanced by IFN survival signals. Our results reveal a novel mechanism underlying the infection-induced apoptosis that can be modulated by the NS1 and type I IFN signaling in IAV-infected cells.

## 1. Introduction

Host cells have developed various defenses against influenza A virus (IAV) infection. The most important defense mechanism is the innate response of production of type I interferons (IFNs), which induce an antiviral state in infected cells and neighboring uninfected cells by triggering the expression of IFN-inducible genes or IFN-stimulated genes [1,2,3]. In addition, host cells can limit IAV replication by inducing apoptosis of infected cells [4,5]. Despite the strong antiviral defenses of host cells, IAVs still replicate in host cells because they have evolved strategies to thwart host antiviral defenses. The non-structural protein 1 (NS1) of IAVs is a master in this regard and facilitates IAV replication by inhibiting both IFN induction and apoptosis in infected cells [2,3,6]. The NS1 protein suppresses IFN expression through the inhibition of retinoic acid-inducible gene (RIG-I)-mediated activation of the transcription factors interferon regulatory factor-3 (IRF-3), activating protein–1 (AP-1), and NF-kB that target the IFN promoter [7,8]. The NS1 protein also suppresses IFN expression by inhibiting the maturation and nuclear export of host mRNAs, including IFN mRNAs [9,10]. One of the major mechanisms by which the NS1 inhibits apoptosis is to activate the PI3K/Akt pathway via binding the p85 subunit of PI3K [11,12].

RuvB (bacterial RuvB gene/protein)-like proteins 1 and 2 (RuvBL1 and RuvBL2) are putative ATPases and belong to the AAA+ (ATPase associated with diverse cellular activities) family of ATPase [13,14,15]. In addition to the ATPase activity, they contain helicase activity that unwinds DNAs and RNAs [16]. In addition, the chaperone activity of the proteins enables them to play important roles in the assembly and remodeling of protein–protein and protein–DNA/RNA complexes [13,14,17]. RuvBL1 and RuvBL2 consist of 456 and 463 amino acids, respectively, share 43% identity, can form hetero-complexes, and may function together or individually [16,18,19]. They are conserved in evolution and play important roles in a variety of cellular processes, such as chromatin remodeling, transcription regulation, and DNA repair [13,14,20,21,22]. It has been shown that RuvBL1 and/or RuvBL2 are involved in cancer [23,24,25] and replication or other aspects of different viruses, including IAVs [26,27], HIV [28], Ebola virus [29], West Nile virus [30], adenovirus [31], and Hepatitis B virus [32].

In the present study, we identified RuvBL1 and RuvBL2 as interacting partners of the NS1 protein of influenza A/PR/8/34 (PR8) virus. Infection of human lung A549 cells with PR8 virus resulted in a reduction of the protein levels of RuvBL2 but not RuvBL1. Further studies with RuvBL2 demonstrated that the NS1-RuvBL2 interaction is RNA-independent, and RuvBL2 binds the RNA-binding domain (RBD) of the NS1. PR8 virus induces apoptosis in infected cells by suppressing the protein levels of RuvBL2 in the absence of IFN influence, and the NS1 protein inhibits infection-induced apoptosis by maintaining RuvBL2 protein abundance. In addition, we show that the viral infection-induced, RuvBL2-regulated apoptosis is counterbalanced by the survival signals of type I IFNs in infected cells.

## 2. Materials and Methods

### 2.1. Cell Culture, Virus Infection, and Plasmid Preparation

Human lung epithelial A549 cells and African green monkey kidney Vero cells were purchased from the American Type Culture Collection (ATCC, Manassas, VA, USA) and maintained in Dulbecco modified eagle medium (DMEM; Invitrogen, Carlsbad, CA, USA) supplemented with 10% fetal bovine serum (FBS; Atlanta Biologicals, Lawrenceville, GA, USA) and 1% penicillin and streptomycin. Wild-type (WT) PR8 virus was purchased from ATCC, propagated, and titrated, as described previously [33]. delNS1 influenza PR8 virus was kindly provided by Dr. Adolfo Garcia-Sastre (Icahn School of Medicine at Mount Sinai, New York, NY, USA) [34], propagated, and titrated in Vero cells as WT virus. The DNA sequence coding for the IAV H7N9 NS1 protein from the pCX-V5-H7N9-NS1 plasmid [35] was cloned into the vector pcDNA 3.1 for the expression of the NS1 in 293T cells. The plasmids for the expression of other genes/gene regions are described in other sections.

### 2.2. Two-Dimensional Gel Electrophoresis (2-DE), LC-MS/MS Analysis, and Database Search

We used a 2-DE-based proteomic approach to identify cellular proteins that interacted with the NS1 protein. Briefly, two populations of 293T cells were transiently transfected with plasmids that expressed Flag alone (control) or Flag-NS1, respectively. After affinity purification of the cell lysates from the two populations of cells with immobilized anti-Flag antibodies, the bound proteins were fractionated by 2-DE, as described previously [33,36,37]. The protein spots uniquely appearing in the gel that resolved the proteins purified from the cells that expressed Flag-NS1 were excised, in-gel digested, and the resulting peptides were analyzed by LC-MS/MS.

LC-MS/MS analysis was performed as described previously [36,37,38]. Briefly, proteins in 2-D gel spots were digested with trypsin (Promega, Madison, WI, USA) overnight at 37 °C and the resulting peptides were dissolved in 20 μL 0.1% formic acid, and analyzed with an LTQ-XL mass spectrometer (Thermo, San Jose, CA, USA) at the Proteomic Facility at the University of Arkansas for Medical Sciences (Little Rock, AR, USA), as described [38]. The raw data were converted into peak lists in mgf files with ProteoWizard 3.0.7665 through Mascot Daemon (2.5.1; Matrix Science, London, UK). Mascot was used to search against a composite target-decoy SwissProt database taxonomic field for human (May 2019, 20,432 entries) or virus (May 2019, 16,647 entries) using the mgf files. The parameters for database searching were as follows: 2.0-Da mass error tolerance for MS and 0.5 Da for MS/MS, tryptic enzyme specificity with a maximum of 2 missed cleavages, fixed modification of carbamidomethyl of cysteine, and variable modifications of acetylation at the peptide N terminus, and oxidation on methionine. Peptide matches with significant homology (*p* < 0.05) were considered to be identified peptides. Search results were further processed by Scaffold software (version 4.9.0; Proteome Software, Portland, OR, USA) to validate MS/MS-based peptide and protein identifications. Peptide identifications were accepted if they could be established at greater than 70.0% probability to achieve a false discovery rate of less than 1.0%. Protein identifications were accepted if they could be established at greater than 99.0% probability to achieve a false discovery rate of less than 1.0% and contained at least 2 identified peptides.

### 2.3. Immunoprecipitation (IP)

The HA-tagged RuvBL1 or RuvBL2 were inserted into the pCruz vector (Santa Cruz Biotech, Santa Cruz, CA, USA), and the Flag-tagged NS1 of PR8 virus was inserted into pcDNA 3.1 for expression in 293T cells. For co-IPs, lysates from the cells expressing Flag-NS1 and HA-RuvBL2/HA-RuvBL1, and the cells expressing Flag alone and HA-RuvBL2/HA-RuvBL1 were immunoprecipitated with an immobilized anti-Flag antibody. After washing, the bound proteins were eluted by boiling in SDS-PAGE loading buffer and analyzed with Western blotting using an anti-HA antibody. For reciprocal co-IPs, lysates from the cells expressing Flag-NS1 and HA-RuvBL2/HA-RuvBL1, and the cells expressing HA alone and Flag-NS1 were immunoprecipitated with an immobilized anti-HA antibody. After washing, the bound proteins were eluted by boiling in SDS-PAGE loading buffer and analyzed with Western blotting using an anti-Flag antibody. Transfection and IPs were performed as described previously [37]. An RNase A treatment (0, 5, or 50 µg/mL of RNase A at 4 °C for 1 h) in IPs was performed on immunoprecipitated proteins on beads before washing. After washing, the bound proteins were eluted by a buffer containing 3× Flag peptide and analyzed with Western blotting using the indicated antibodies. In the Western blot analysis, the amount of each Input sample loaded onto the SDS-PAGE gel was 1% of the cell lysate used for the IP.

### 2.4. Protein Expression and In-Vitro Binding Assay

The DNA sequences encoding GST, GST-tagged full-length NS1, the GST-tagged RBD (amino acids 1–73), or the effector domain (ED) (amino acids 85–230) of the NS1 of PR8 virus were cloned in the vector pGEX-6p-2, and the sequences encoding Flag-tagged human RuvBL2 were cloned in the vector pET-28. The recombinant proteins were expressed in *E. coli*, as described previously [36]. Equal molar amounts of purified GST-NS1 (GST, GST-RBD, or GST-ED) and Flag-RuvBL2 were mixed and incubated in a binding buffer (10 mM Tris-HCl, pH 7.5, 50 mM NaCl, 1 mM EDTA, 1.5 mM MgCl_2_, 0.1% Triton X-100, and protease inhibitors) for 2.5 h at 4 °C with end-to-end rotation. Then, 15 μL of glutathione agarose resin (Gold Biotechnology, St. Louis, MO, USA) were added to the mixtures and the mixture was incubated for an additional 1.5 h at 4 °C with end-to-end rotation. After washing 3 times with a wash buffer (10 mM Tris-HCl, pH 7.5, 150 mM NaCl, 1 mM EDTA, 1.5 mM MgCl_2_, and 0.1% Triton X-100), the bound proteins were eluted with an elution buffer (50 mM Tris-HCl, pH 8.0, and 10 mM reduced glutathione) and examined by Western blotting.

### 2.5. siRNAs and Overexpression

Two siRNA sequences targeting RuvBL2 (5′-GAGACCAUCUACGACCUGGGCAC-3′ and 5′-GAGAGUGACAUGGCGCCUGUCCU-3′) [39] were co-transfected into Vero cells to knock down the expression of RuvBL2 as described previously [33]. A randomized siRNA sequence was used as a control [37]. For RuvBL2 overexpression, the coding sequence of RuvBL2 was inserted into pcDNA3.1 plasmid, and the resulting construct was transfected into Vero cells using Lipofectamine LTX PLUS (Invitrogen, Carlsbad, CA, USA) according to the manufacturer’s instructions.

### 2.6. Western Blotting

Western blotting was performed as described previously [33,37]. Antibodies against RuvBL1, beta-actin, and Annexin I were purchased from Santa Cruz Biotech (Santa Cruz, CA, USA). Antibodies against RuvBL2 and poly(ADP-ribose) polymerase (PARP) were from BD (San Jose, CA, USA). A mouse monoclonal anti-NS1 antibody was kindly provided by Dr. Stephan Ludwig (University of Muenster, Muenster, Germany).

### 2.7. Caspase 3/7 Assay

The activities of caspase 3/7 were determined with the Caspase-Glo 3/7 kit (Promega, Madison, WI, USA) according to the manufacturer’s instructions. Briefly, the treated Vero cells or A549 cells in 96-well plates were incubated with caspase reagent provided by the kit at room temperature for 1 h, and the luminescence was then measured by a spectrofluorometer (SpectraMax Gemini XS, Molecular Devices). All analyses were performed with 3 separate sample preparations.

### 2.8. Pretreatment of Vero Cells with Recombinant IFN-α

Vero cells were cultured in DMEM supplemented with 10% FBS in 6-well plates overnight, and the culture medium was replaced with fresh medium containing the recombinant universal human IFN-αA/D (rHuIFN-αA/D; PBL Biomedical Labs, Piscataway, NJ, USA) at 1000 units mL^−1^ [40,41] the next morning. After incubation with IFN-α for 6 h, the cells were infected with WT or delNS1 virus at an MOI of 1 and harvested at 36 and 48 hpi for analysis.

### 2.9. Statistical Analysis

The Student’s *t*-test was used to assess statistical significance. Data were expressed as mean ± SE. Differences were tested by a two-tailed *t*-test. Differences in mean values were considered significant when *p* was ≤0.05.

## 3. Results

### 3.1. Identifications of the Proteins That Potentially Interact with the NS1 Protein

**Figure 1:**
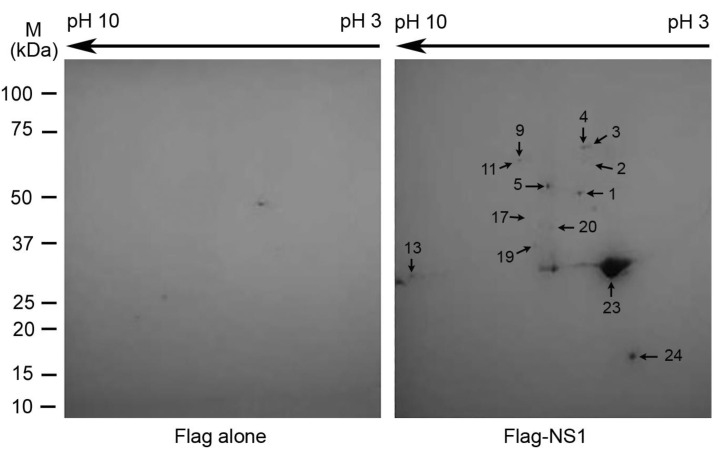
Two-dimensional gel fractionation of the affinity-purified proteins. The Flag-NS1 and its associated proteins were pulled down by immobilized anti-Flag antibody and fractionated with 2-DE. The fractionations of the proteins pulled down from the lysate of the cells expressing Flag alone (control, left panel), and the cells expressing Flag-NS1 (right panel) are shown. The prominent proteins identified in the indicated protein spots by LC-MS/MS are listed in Table 1.

A 2-DE-based proteomic method [36,37] was used to identify cellular proteins associated with the NS1 protein of PR8 virus. The results revealed that multiple protein spots appeared in the “NS1 gel” (the gel that resolved the proteins pulled down from the lysate of the cells expressing the NS1) but not in the control gel (the gel that resolved the proteins pulled down from the lysate of the cells expressing Flag alone) (Figure 1). LC-MS/MS analysis of the proteins in those spots and a subsequent database search against the SwissProt human database revealed that multiple proteins were unambiguously identified (Table 1). Two of the most abundant proteins that were pulled down with the Flag-NS1 were RuvBL1 and RuvBL2 (Table 1), which were also the two largest protein spots in the 2-D gel (Figure 1, Spots 1 and 5) except for the bait protein (Spots 23 and 24; see below). The two proteins were identified with high confidence by LC-MS/MS: 22 and 25 peptides unique to RuvBL1 and RuvBL2, respectively. RuvBL1 and RuvBL2 are members of the AAA+ family of helicase [14,15] and have been shown to play important roles in a variety of cellular activities [13,20]. In relation to IAV infection, Mayer et al. (2007) and Kakugawa et al. (2009) reported that RuvBL2 interacted with influenza A viral ribonucleoproteins (vRNPs) [26,27] and inhibited the viral polymerase via disrupting the assembly of NP protein oligomers [26].

Multiple other proteins were also identified to potentially interact with the NS1 (Table 1). The remaining proteins were members of either the heterogeneous nuclear ribonucleoprotein (hnRNP) family or the heat-shock protein family (Table 1). hnRNP A2/B1 (Figure 1, Spot 13) plays important roles in RNA processing [42], and has previously been shown to interact with the NS1 and inhibit IAV replication [37]. We also searched the SwissProt protein database taxonomic field for viruses using the LC-MS/MS data to reveal the bait protein NS1. As expected, the NS1 (Spot 23) was the most abundant protein in the pulled-down proteins. The bait protein NS1 was so abundant that it could not be well-separated by isoelectric focus in the 2-DE (Figure 1; Figure S1 and Table S1). Interestingly, besides the full-length NS1 (Spot 23), a much less-abundant and shorter version of the NS1 was also revealed by 2-D gel (Spot 24). At present, it is not clear whether the shorter NS1 was truncated in vivo under physiological conditions or in vitro in the processes of cell lysis preparation, affinity purification, and/or 2-DE. If it is the former, it would be interesting to understand whether the truncated version plays any role in IAV replication. In addition to the top-ranked proteins with the highest spectrum count in each protein spot in the 2-D gel, which are listed in Table 1, typically several other proteins with lower spectrum counts were also identified in each spot. Furthermore, some proteins were identified in more than one protein spot. The complete list of the identified proteins, including those lower-ranked proteins in each spot and the proteins identified in fainter protein spots, were listed in Table S1, and the corresponding 2-D gel spots are shown in Figure S1. From the identified proteins (Table S1), it is clear that we were unable to identify some of the proteins that have been shown to interact with the NS1, such as PI3K [12], DDX21 [43], TRIM25 [44], and RIG-1 [7,8,45]. The lack of those proteins in the present identification likely resulted from the limitation of the 2-DE, which has limited capacities in resolving low-abundant proteins, large proteins, membrane proteins, and proteins with high or low isoelectric points [46]. Because RuvBL1 and RuvBL2 were abundantly associated with the NS1 (Table 1 and Figure 1), and RuvBL2 has been shown to affect IAV infection [26,27], we selected these two proteins for further characterization of their interactions with the NS1 and their potential roles in IAV infection.

### 3.2. RuvBL1 and RuvBL2 Interact with the NS1

**Figure 2:**
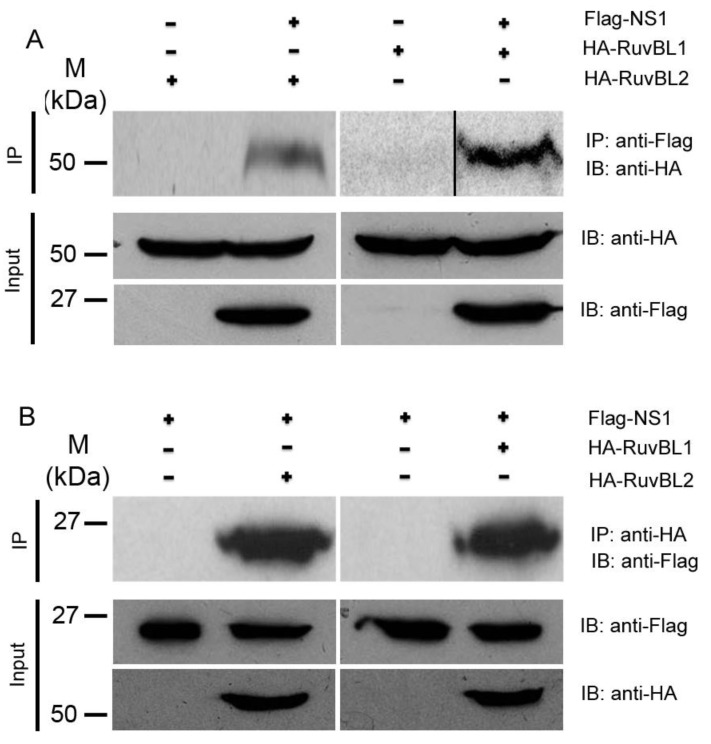
Validation of the interactions between the NS1 and RuvBL1/RuvBL2. (**A**) co-IPs: Cell lysates from the cells expressing Flag-NS1 and HA-RuvBL1/HA-RuvBL2, or the cells expressing Flag alone and HA-RuvBL1/HA-RuvBL2 (control), were immunoprecipitated with anti-Flag M2 resin, and the immunoprecipitated proteins were probed with an anti-HA antibody in Western blotting. (**B**) Reciprocal co-IPs, cell lysates from the cells expressing Flag-NS1 and HA-RuvBL1/HA-RuvBL2, or the cells expressing HA alone and Flag-NS1 (control), were precipitated with an immobilized anti-HA antibody, and the immunoprecipitated proteins were probed with an anti-Flag antibody in Western blotting. IP: immunoprecipitation; IB: immunoblotting.

To validate the interactions between the NS1 protein and RuvBL1/RuvBL2, we performed reciprocal co-IPs [37,47]. While an immobilized anti-Flag antibody precipitated significant amounts of HA-RuvBL1 and HA-RuvBL2 from the cells that expressed Flag-NS1 and HA-RuvBL1 or HA-RuvBL2, respectively, the antibody did not precipitate detectable levels of HA-RuvBL1 and HA-RuvBL2 from the cells that expressed Flag alone and HA-RuvBL1 or HA-RuvBL2 (Figure 2A). Similarly, in the reciprocal co-IPs, an immobilized anti-HA antibody precipitated large amounts of Flag-NS1 from the cells that expressed Flag-NS1 and HA-RuvBL1 or HA-RuvBL2, whereas the immobilized anti-HA antibody failed to precipitate any detectable amount of Flag-NS1 from the cells that expressed Flag-NS1 and HA tag alone (Figure 2B).

**Figure 3:**
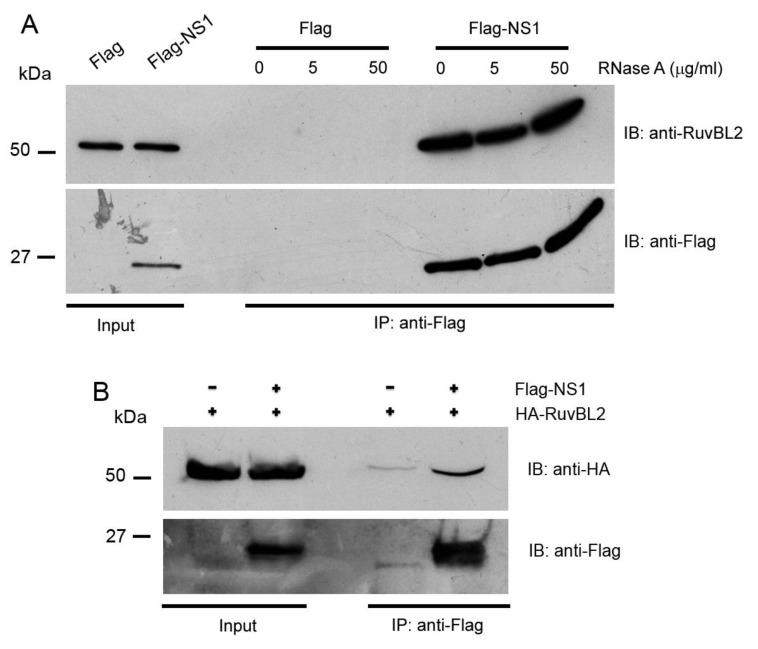
The NS1 interacts with endogenous RuvBL2 and the NS1-RuvBL2 interaction is RNA-independent. (**A**) The NS1 interacts with endogenous RuvBL2 and the NS1-RuvBL2 interaction is RNA-independent. Cell lysates of the cells expressing Flag alone and an endogenous RuvBL2, or the cells expressing Flag-NS1 and an endogenous RuvBL2 were pulled down by an immobilized anti-Flag antibody, and the precipitated proteins on beads were treated with the indicated concentrations of RNase A, washed, and the bound proteins were analyzed by Western blotting with the indicated antibodies. (**B**) RuvBL2 interacts with the NS1 of the seasonal IAV H7N9 virus. The experiment was performed similarly to that shown in Figure 2A.

Because our subsequent functional studies showed that IAV infection affected protein levels of RuvBL2 but not RuvBL1 (see below), we focused further protein–protein interaction studies and functional studies on RuvBL2. The NS1 and RuvBL2 are both RNA-binding proteins; we first examined whether the NS1-RuvBL2 interaction was RNA-dependent. For this purpose, we pulled down the NS1 and its associated proteins with immobilized anti-Flag antibody from the lysate of the cells expressing Flag-NS1 (Flag alone for control) and endogenous RuvBL2, treated the bound proteins on beads with RNase A, washed the beads, eluted the bound proteins with 3X Flag peptide, and analyzed the bound proteins with Western blotting. The results demonstrated that RNase A digestion did not affect the interaction between the NS1 and endogenous RuvBL2 (Figure 3A), suggesting that the NS1-RuvBL2 interaction is not RNA-dependent. To test whether RuvBL2 interacts with the NS1 of seasonal influenza A viruses, we ectopically expressed Flag-NS1 (Flag alone for control) of a 2013 H7N9 virus (strain A/Zhejiang/DTID-ZJU01/2013) [35] in 293T cells, pulled down the NS1 and its associated proteins, and analyzed the bound proteins with Western blotting. The results demonstrated that immobilized anti-Flag antibody pulled down more HA-RuvBL2 from the lysate of Flag-NS1-expressing cells than from the lysate of Flag alone-expressing cells (Figure 3B), suggesting that the NS1-RuvBL2 interaction is not restricted to the NS1 of PR8 viruses but is shared by the NS1 of other IAVs.

### 3.3. RuvBL2 Physically Binds the RNA-Binding Domain (RBD) of the NS1

**Figure 4:**
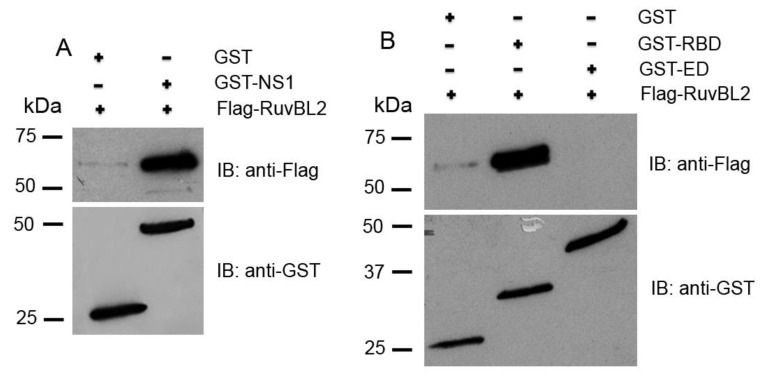
RuvBL2 binds the RBD domain of the NS1 protein. (**A**) RuvBL2 physically binds the full-length NS1. (**B**) RuvBL2 physically binds the RBD domain of the NS1. Purified GST, GST tagged full-length NS1, GST-ED, or GST-RBD was incubated with equal molar amounts of purified Flag-RuvBL2, followed by pulldown by glutathione beads. The bound proteins were analyzed by Western blotting.

To further assess whether the NS1 directly binds RuvBL2, we expressed Flag-tagged RuvBL2 and the GST-tagged full-length NS1 of PR8 virus in *E. coli*, purified them with affinity purification, and performed in vitro GST pulldown assays. The results demonstrated that the NS1 physically bound RuvBL2 (Figure 4A, upper panel). The NS1 protein is composed of an N-terminal RBD domain and a C-terminal effector domain (ED) linked by a short linker [2,3]. To determine which domain of the NS1 binds RuvBL2, we expressed the GST-RBD domain (amino acids 1–73), GST-ED domain (amino acids 85–230) of the NS1 of PR8 virus, and Flag-RuvBL2 in *E. coli*, purified them, and performed in vitro GST pulldown assays. The results demonstrated that RuvBL2 bound the RBD domain but not the ED domain of the NS1 of PR8 virus (Figure 4B, upper panel). We confirmed that appropriate amounts of GST, GST-ED, GST-RBD, and GST-NS1 were used in the pulldown assays (Figure 4, lower panels).

### 3.4. IAV Infection Leads to Reductions in RuvBL2 Protein Abundance in Infected Cells, and the NS1 Inhibits the Infection-Induced Reduction in RuvBL2 Abundance

**Figure 5:**
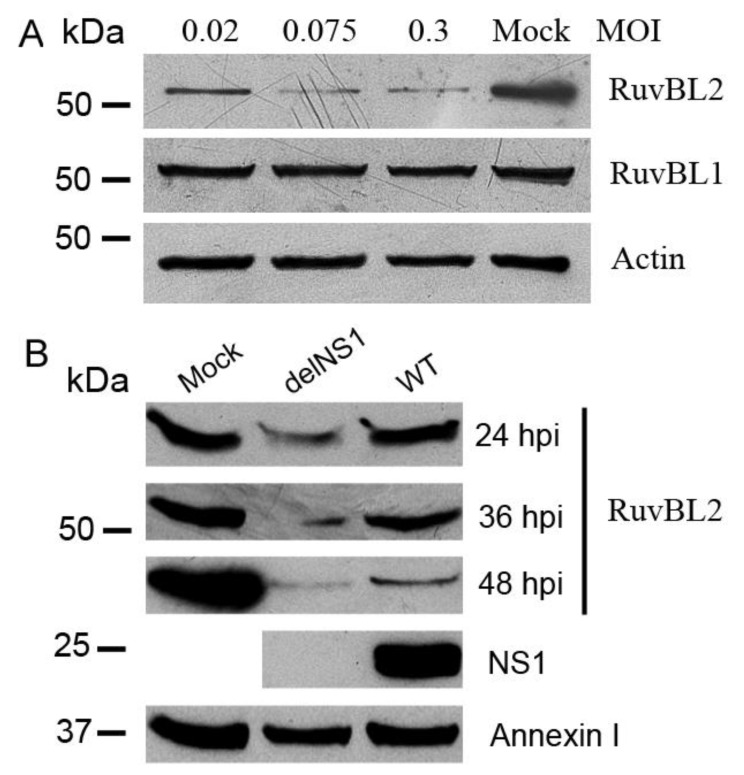
IAV infection suppresses cellular RuvBL2 protein abundance, and the NS1 protein acts to maintain RuvBL2 protein abundance. (**A**) IAV virus infection suppresses the protein levels of RuvBL2 but not RuvBL1 in A549 cells. A549 cells were mock-infected or infected with WT PR8 virus at the indicated MOIs for 36 h and the lysates were analyzed by Western blotting with the indicated antibodies. β-actin was used as a loading control. (**B**) IAV infection suppresses the protein levels of RuvBL2 in Vero cells and the NS1 protein acts to maintain RuvBL2 protein levels in infected cells. Vero cells were mock-infected or infected by WT or delNS1 PR8 virus at an MOI of 1 for 24, 36, or 48 h, and the cell lysates were then analyzed by Western blotting with the indicated antibodies. Annexin-1 was used as a loading control [38].

To assess the potential role of the interactions between the NS1 and RuvBL1 or RuvBL2 proteins in IAV replication, we infected A549 cells with WT PR8 virus at MOIs of 0.02, 0.075, and 0.3 (or mock-infected for control) for 36 h, and then examined the protein levels of RuvBL1 and RuvBL2 in infected cells with Western blotting. The results demonstrated that RuvBL2 protein levels in infected cells were reduced by PR8 virus infection. In contrast to RuVBL2, the infection had no apparent effect on RuvBL1 protein levels (Figure 5A). To assess whether the NS1 protein is involved in regulating RuvBL2 protein abundance in infected cells, we infected Vero cells, which are type I IFN-deficient [48], with either WT or delNS1 [34] PR8 virus at an MOI of 1 and harvested the cells at 24, 36, and 48 hpi for Western blot analysis. The results demonstrated that RuvBL2 protein levels were suppressed by both types of viruses in Vero cells, but delNS1 virus resulted in more pronounced reductions in RuvBL2 protein levels than did WT virus (Figure 5B). As expected, delNS1 virus-infected cells did not contain the NS1 protein (Figure 5B). These results suggest that (1) PR8 virus infection suppresses RuvBL2 protein abundance in infected cells, and a non-NS1 viral element or elements in PR8 virus are responsible for the reduction of RuvBL2 protein levels, and (2) the NS1 protein antagonizes the non-NS1 viral element-induced reduction in RuvBL2 protein abundance in infected cells.

### 3.5. IAV Infection Induces Host Cell Apoptosis by Suppressing Cellular RuvBL2 Protein Abundance

**Figure 6:**
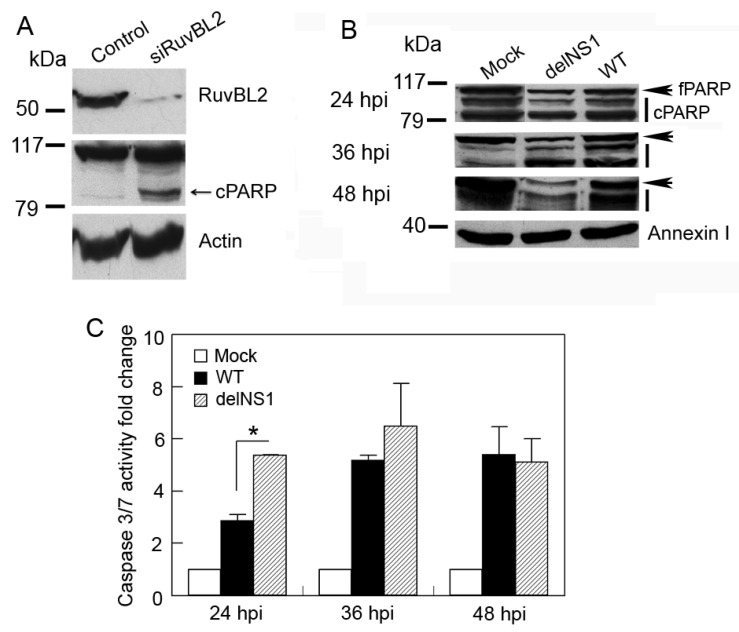
Knockdown of RuvBL2 induces apoptosis in Vero cells. (**A**) Knockdown of RuvBL2 by siRNAs leads to increased PARP cleavage in Vero cells. (**B**,**C**) IAV infection induces apoptosis in Vero cells, and the NS1 protein acts to inhibit virus-induced apoptosis in Vero cells. Vero cells were mock-infected or infected by WT or delNS1 PR8 virus at an MOI of 1 for 24, 36, and 48 h and then harvested for analysis by Western blotting with the indicated antibodies (**B**) or determination of caspase 3/7 activities (**C**). Values in (**C**) are the means ± SE of three separate sample preparations. * *p* < 0.05. Actin and annexin I in (**A**,**B**) were used as loading controls. fPARP, full-length PARP; cPARP, fragments of cleaved PARP.

**Figure 7:**
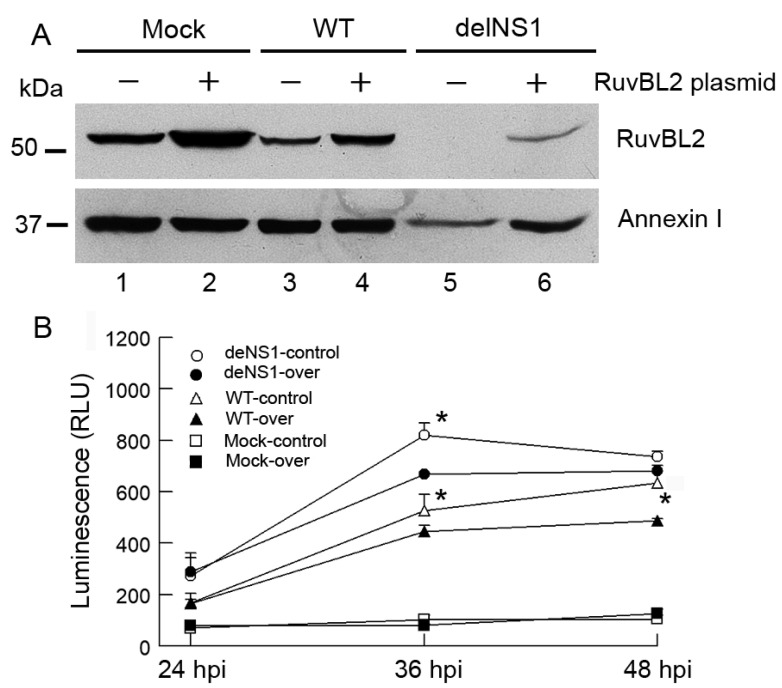
Overexpression of RuvBL2 promotes resistance to infection-induced apoptosis. Vero cells were transfected with an empty vector (control) or a plasmid that expressed RuvBL2, and the transfected cells were then mock-infected or infected with WT or delNS1 PR8 virus at an MOI of 1. The cells were either harvested at 36 hpi for Western blot analysis (**A**), or at the indicated time points for measurements of caspase 3/7 activities (**B**). Values in (**B**) are the means ± SE of three separate sample preparations. * denotes *p* < 0.05 and indicates the statistical differences between the cells that overexpressed RuvBL2 (over) and the corresponding control cells that did not overexpress RuvBL2 (control).

To examine the biological consequences of IAV-induced reduction in RuvBL2 protein abundance in infected cells, we used small interfering (siRNAs) to knock down RuvBL2 expression and examined the effect of the knockdown on viral protein synthesis, virus replication, and apoptosis. The results demonstrated that knockdown of RuvBL2 in Vero cells had no significant effect on the NS1 protein levels and PR8 virus replication (data not shown). However, knockdown of RuvBL2 induced apoptosis in Vero cells (Figure 6A). The result is consistent with a previous report, which demonstrated that knockdown of RuvBL2 by siRNAs induced apoptosis in human liver cancer cells [24]. To determine how the NS1 protein affects RuvBL2-regulated apoptosis in infected cells, we infected Vero cells with WT or delNS1 PR8 virus at an MOI of 1 and examined apoptosis at 24, 36, and 48 hpi. The results demonstrated that both WT and delNS1 viruses induced apoptosis, evidenced by increased cleavage of PARP (Figure 6B) and the activations of caspase 3/7 (Figure 6C, fold changes: 2.9–6.5), and delNS1 virus induced more apoptosis than did WT virus (Figure 6B,C at 24 hpi). This result coincides with the results shown in Figure 5B, which shows that delNS1 virus induced more reductions in RuvBL2 protein levels than did WT virus, suggesting that IAVs might induce apoptosis by suppressing RuvBL2 protein abundance in infected Vero cells. To test this possibility, we overexpressed RuvBL2 in Vero cells by transiently transfecting the cells with a plasmid that expressed RuvBL2 or empty vector for control, followed by infection of the transfected cells with WT or delNS1 PR8 virus at an MOI of 1 (mock infection for control) 24 h after the transfection. The transfected/infected cells were harvested at 36 hpi for Western blot analysis, or 24, 36, and 48 hpi for caspase 3/7 activity determinations. The results demonstrated that, like what was observed in the untransfected cells (Figure 5B), infection with WT or delNS1 PR8 virus reduced the protein levels of RuvBL2 in the transfected cells regardless if RuvBL2 was overexpressed or not (Figure 7A, compare lanes 3 and 5 with lane 1, and compare lanes 4 and 6 with lane 2), and delNS1 virus infection resulted in more reductions in RuvBL2 protein levels than did WT virus (Figure 7A, compare lane 5 with lane 3 in reference to lane 1; compare lane 6 with lane 4 in reference to lane 2). Importantly, the cells that overexpressed RuvBL2 were more resistant to WT or delNS1 virus-induced apoptosis than the corresponding control cells that did not overexpress RuvBL2 at 36 and 48 hpi (Figure 7B; except for the delNS1-infected cells at 48 hpi). These results suggest that IAV infection induces apoptosis by suppressing RuvBL2 abundance in infected Vero cells.

### 3.6. Type I IFNs Counteract the Viral Infection-Induced, RuvBL2-Regulated Apoptosis

**Figure 8:**
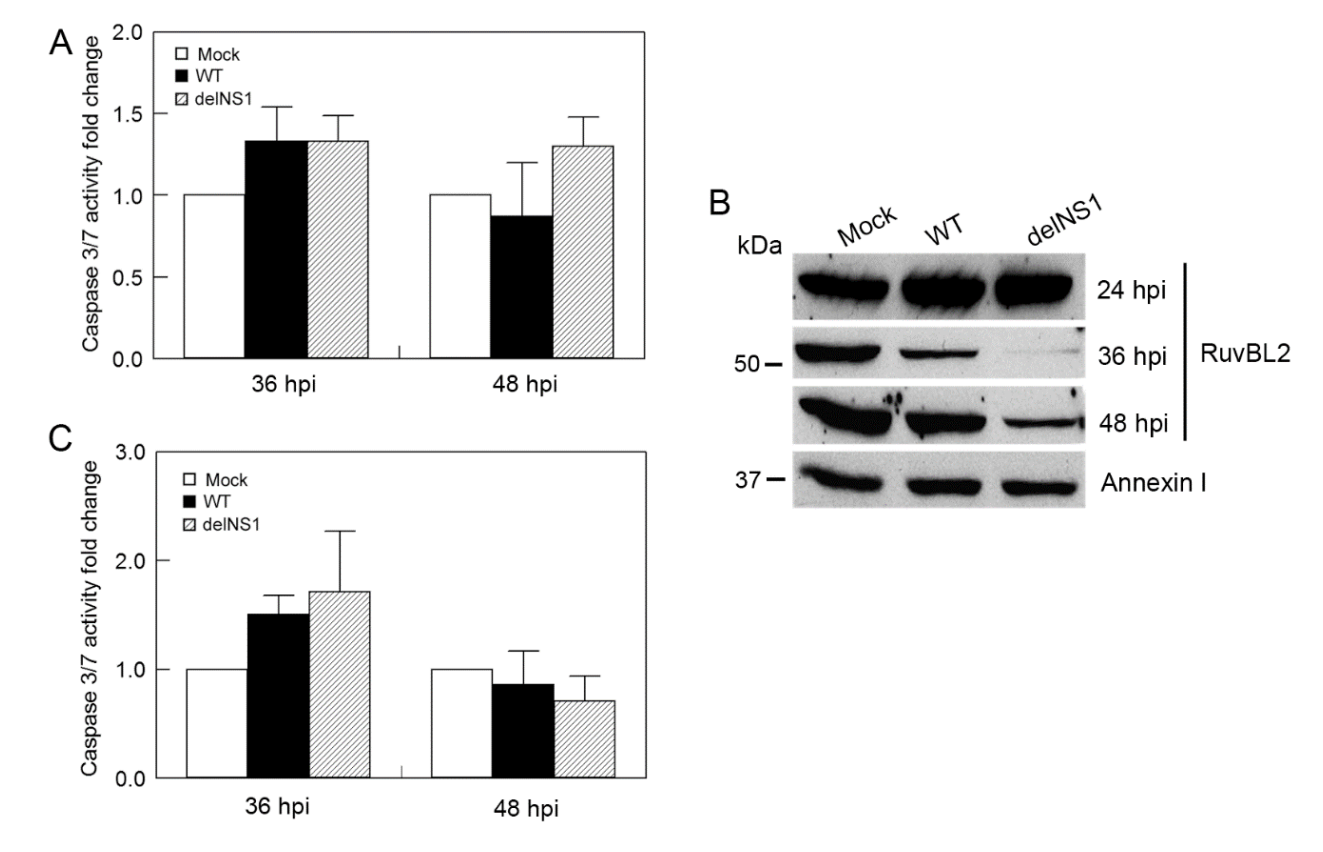
IFNs inhibit IAV-induced apoptosis in infected cells. (**A**) IAV infection does not induce apoptosis in A549 cells. A549 cells were mock-infected or infected with WT or delNS1 PR8 virus at an MOI of 1 for 36 and 48 h, and then harvested for determinations of caspase 3/7 activities. (**B**,**C**) IAV infection suppresses RuvBL2 cellular protein abundance but does not affect apoptosis in the Vero cells pretreated with a recombinant IFN. Vero cells were pretreated with a recombinant universal human IFN-α at 1000 U mL^−1^ for 6 h, and then mock-infected or infected with WT or delNS1 virus at an MOI of 1. The cells were harvested at the indicated time points either for Western blotting analysis (**B**) or caspase 3/7 activity measurement (**C**). Values in (**A**,**C**) are the means ± SE of three separate sample preparations.

We also tested the effect of WT and delNS1 PR8 viruses on apoptosis in IFN-competent A549 cells. In contrast to IFN-deficient Vero cells, in which WT or delNS1 PR8 virus infection increased caspase 3/7 activity by approximately 6-fold at 36 and 48 hpi (Figure 6C), infection of A549 cells with WT or delNS1 virus at the same MOI (an MOI of 1) increased the activity less than 1.5-fold at 36 and 48 hpi (Figure 8A), suggesting that the type I IFNs inhibit apoptosis in infected cells. To test this notion, we pretreated Vero cells with a recombinant universal human IFN-α and infected the pretreated cells with WT or delNS1 PR8 virus at an MOI of 1, and harvested the infected cells at 24, 36, and 48 hpi for Western blot analysis and caspase activity determination. We found that both WT and delNS1 viruses reduced RuvBL2 protein levels in infected cells at 36 and 48 hpi (Figure 8B) but did not induce significant apoptosis (Figure 8C; fold changes: 0.9–1.8), which was comparable to what was observed in A549 cells (Figure 8A). These results contrast sharply with what was observed in Vero cells without IFN pretreatment (Figure 6B,C), which demonstrated that WT or delNS1 virus infection induced substantial increases in apoptosis in infected cells at 36 hpi and 48 hpi (fold changes at 36 hpi and 48 hpi in Figure 6C: 5.2–6.5). The contrast suggests that type I IFNs counterbalance the RuvBL2-regulated apoptosis in infected cells.

## 4. Discussion

In the present study, we identified RuvBL1 and RuvBL2 as interacting partners of the NS1 protein of IAVs (Figure 1 and Table 1). We verified the interactions by reciprocal co-IPs (Figure 2) and demonstrated that the NS1 interacts with endogenous RuvBL2 and the NS1-RuvBL2 interaction was not RNA-dependent (Figure 3A). Furthermore, we showed that RuvBL2 physically bound the RBD domain of the NS1 protein (Figure 4). Functional studies demonstrated that delNS1 PR8 virus induced more reductions in RuvBL2 protein levels than did WT virus (Figure 5B), suggesting that a non-NS1 viral element or elements are responsible for the reduction in RuvBL2 abundance in infected cells, and NS1 acts to maintain RuvBL2 protein abundance by antagonizing the non-NS1 element-induced reduction in RuvBL2 protein abundance in infected cells. Knockdown of RuvBL2 in Vero cells by siRNAs induced apoptosis (Figure 6A) and overexpression of RuvBL2 resulted in increased resistance to IAV-induced apoptosis (Figure 7), suggesting that IAV infection induces apoptosis by suppressing RuvBL2 protein abundance in infected cells. Further studies demonstrated that the viral infection-induced, RuvBL2-regulated apoptosis in IAV-infected cells was overwhelmed by IFN signals (Figure 6 and Figure 8), which suggests that RuvBL2 and IFNs may regulate apoptosis in IAV-infected cells through a shared pathway.

**Figure 9:**
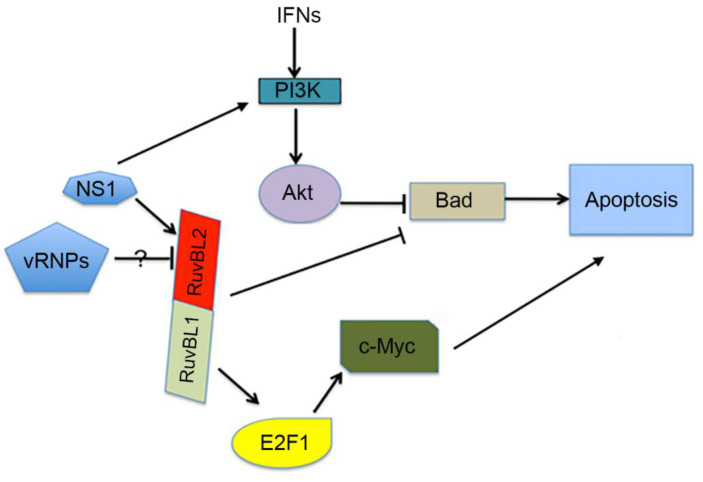
A proposed model underlying the regulation of apoptosis by RuvBL2 in IAV-infected cells. IAV infection suppresses cellular RuvBL2 protein abundance via a non-NS1 viral element(s) (potentially vRNPs), and reduction in RuvBL2 protein abundance induces apoptosis potentially through the upregulation of Bad expression [24]. Type I IFNs counterbalance the infection-induced, RuvBL2-regulated apoptosis in IAV-infected cells by activating the PI3K/Akt pathway [53,54], which, in turn, inactivates Bad [55,56]. The NS1 protein inhibits apoptosis in infected cells through activating the PI3K/Akt pathway by interacting with the p85 subunit of PI3K [12] and by maintaining cellular RuvBL2 protein abundance in IAV-infected cells (present study). IAV infection does not directly affect RuvBL1 protein abundance (Figure 5A). However, RuvBL1 may modulate c-Myc-regulated apoptosis by binding the transcription factor E2F1 [57].

One potential mechanism by which RuvBL2 regulates apoptosis in IAV-infected cells is through affecting the apoptotic activity of the proapoptotic protein Bad (Figure 9). It has been reported that the expression of Bad was upregulated when RuvBL2 was silenced by siRNA [24], suggesting that RuvBL2 negatively regulates Bad expression. Thus, it is possible that IAV infection-induced RuvBL2 reduction can upregulate the expression of Bad, which in turn triggers apoptosis (Figure 9). It is interesting to note that Bad activation is required for efficient IAV replication in host cells [49,50]. However, it is not clear how Bad is activated in IAV-infected cells. Mayer et al. (2007) and Kakugawa et al. (2009) reported that influenza A viral ribonucleoproteins (vRNPs) interacted with RuvBL2 [26,27]. Perhaps the vRNPs could serve as the potential non-NS1 elements that act to suppress RuvBL2 abundance in the infected cells observed in the present study. If this is the case, when vRNPs are accumulated in the nucleus of infected cells, vRNPs can upregulate Bad by suppressing RuvBL2 protein abundance (Figure 9). The upregulated Bad can then facilitate the export of vRNPs from the nucleus to the cytoplasm, presumably by activating other proapoptotic factors such as caspase 3, whose activation is known to also be required for the nuclear export of vRNPs [51]. It was previously thought that IAVs simply take advantage of proapoptotic signaling events, such as the activations of Bad [50] and caspase 3 [51], for efficient IAV replication in infected cells [52]. Based on the present model (Figure 9), it is possible that instead of passively taking advantage of proapoptotic signaling events, IAVs may have evolved a strategy to activate the required molecules (e.g., activated Bad and caspase 3) for the efficient nuclear export of vRNPs when vRNPs are accumulated in adequate amounts in the nucleus of infected cells and are ready for export.

In addition to the antiviral and proapoptotic functions [58], type I IFNs can produce strong cell survival signals to protect cells from apoptosis by activating PI3K and Akt [53,54]. Thus, while IAVs may induce apoptosis in infected cells by downregulating RuvBL2, which in turn upregulates Bad expression [24], type I IFNs can overwhelm the RuvBL2-regulated apoptosis in infected cells by activating PI3K and Akt, which in turn inactivate Bad by phosphorylating Bad at Ser-136 [55,56]. The two pathways may converge on controlling the apoptotic activity of Bad (Figure 9). This proposed model explains why the apoptosis induced by reductions in cellular RuvBL2 abundance occurred only in IFN-deficient Vero cells (Figure 6) but not in IFN-competent A549 cells (Figure 8A), and why IFNs could counterbalance the apoptosis in IAV-infected cells (Figure 8). Because IAVs have evolved several strategies to suppress IFN production in infected cells [7,8,9,10,59], it is possible that the infection-induced, RuvBL2-regulated apoptosis may play a significant role in IAV-induced apoptosis even in IFN-competent cells under certain circumstances. The NS1 protein of IAVs is known to inhibit apoptosis in IAV-infected cells by binding PI3K and activating the PI3K/Akt pathway [11,12]. The results from the present study demonstrate that the NS1 can also inhibit apoptosis in infected cells by maintaining cellular RuvBL2 protein abundance in infected cells (Figure 9). Whether the NS1-RuvBL2 interactions are required for maintaining cellular RuvBL2 protein abundance remains unclear and merits further investigation.

## Figures and Tables

**Table 1:** Short list of proteins identified by affinity purification, 2-DE, and LC-MS/MS analysis ^a^.

Category	Spot # ^b^	Protein Name	SwissProt acc.#	MW (kDa)/pI	Mascot Protein Score	Unique Peptide Count	Exclusive Spectrum Count	Sequence Coverage (%)
ATPase	1	RuvBL2	Q9Y230	51.2/5.5	1859	25	90	53
	5	RuvBL1	Q9Y265	50.2/6.0	1402	22	57	56
Heterogeneous nuclear ribonucleoprotein	2	hnRNP K	P61978	51.0/5.4	1062	17	37	39
	9	hnRNP L	P14866	64.1/8.5	1113	15	25	39
	11	hnRNP Q	O60506	69.6/8.7	675	13	24	18
	13	hnRNP A2/B1	P22626	37.4/9.0	1207	16	33	55
	17	hnRNP D0	Q14103	38.4/7.6	554	5	9	21
	19	hnRNP D-like	O14979	46.4/9.6	499	8	12	16
	20	hnRNP A/B	Q99729	36.2/8.2	362	2	7	11
Heat shock protein	3	Heat shock cognate 71 kDa protein	P11142	70.9/5.4	1566	18	45	32
	4	Heat shock 70 kDa protein 1A/1B	P0DMV8/P0DMV9	70.1/5.5	1395	16	45	32
Influenza A viral protein	23	NS1	P03496	25.9/6.2	1279	18	107	80
	24	NS1	P03496	25.9/6.2	1123	16	68	76

^a^ If two or more proteins were identified in a 2-D gel spot, only the top one was listed in this table. If a protein was identified in two or more spots, only the one with higher or the highest abundance was listed here. Each identified protein contains at least two peptide matches that meet or exceed the threshold values for a 95% confidence level. Refer to Table S1 for the list of all the identified proteins. ^b^ Spot numbers correspond to those in Figure 1.

## Data Availability

The data presented in this study are available in Supplementary Table S1 and Figure S1.

## Supplementary Materials

The following are available online https://www.mdpi.com/article/10.3390/v13061038/s1. Table S1: List of all proteins identified in 2-D gel spots by LC/MS/MS; Figure S1: 2-D gels with all visible protein spots.

## Abbreviations

2-DE	two-dimensional gel electrophoresis
Bad	Bcl-2 antagonist of cell death
delNS1	influenza A virus lacking the gene encoding the non-structural NS1 protein
ED	effector domain
GST	glutathione S-transferase
hnRNP	heterogeneous nuclear ribonucleoprotein
hpi	hours post-infection
IAV	influenza A virus
IB	immunoblotting
IFN	interferon
IP	immunoprecipitation
MOI	multiplicity of infection
NS1	non-structural protein 1
PARP	poly(ADP-ribose) polymerase
PI3K	phosphatidylinositol 3-kinase
RBD	RNA-binding domain
PR8 virus	influenza A/PR/8/34 virus
RuvBL	RuvB-like protein
siRNA	small interfering RNA
vRNP	viral ribonucleoprotein
WT	wild-type
