# Supplementary figures and images for: RuvB-Like Protein 2 Interacts with the NS1 Protein of Influenza A Virus and Affects Apoptosis That Is Counterbalanced by Type I Interferons
Source: Viruses. 2021 May 31;13(6):1038. doi: 10.3390/v13061038 (PMC8229658; doi:10.3390/v13061038)

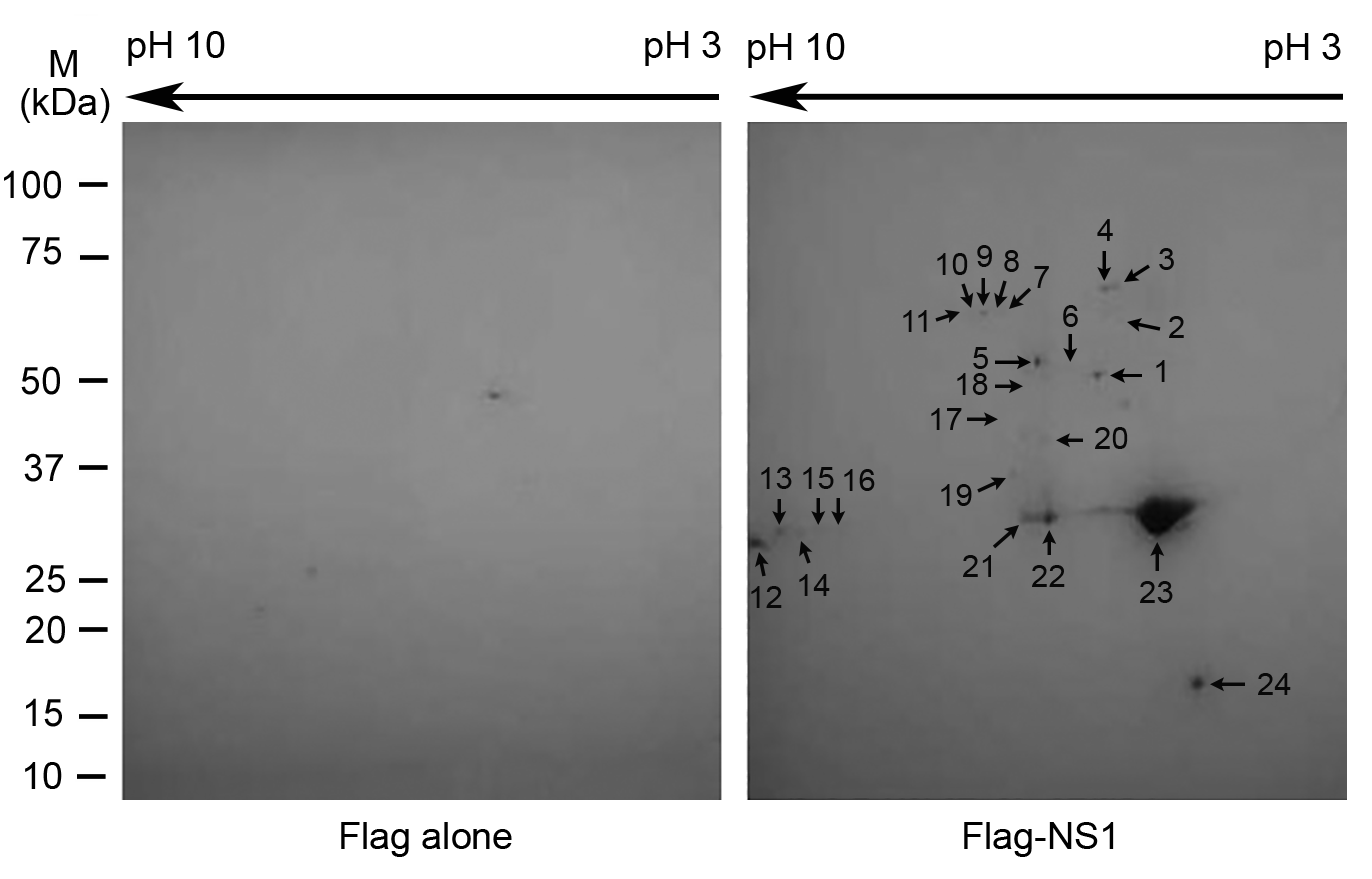

Supplement: Supplementary file 1 [file viruses-13-01038-s001.zip › Figure S1.tif]
